# Supplementary figures and images for: Population-genetic comparison of the Sorbian isolate population in Germany with the German KORA population using genome-wide SNP arrays
Source: BMC Genet. 2011 Jul 28;12:67. doi: 10.1186/1471-2156-12-67 (PMC3199861; doi:10.1186/1471-2156-12-67)

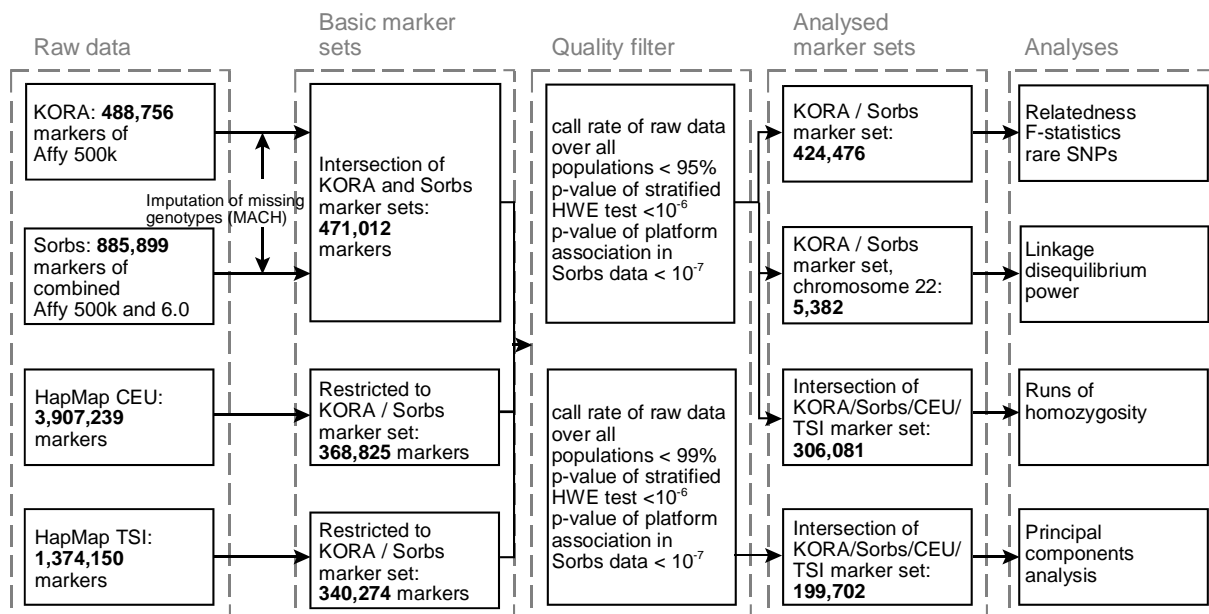

Supplement: Additional file 1 — Workflow of data pre-processing. The workflow of data pre-processing is presented. We start with the autosomal SNP data of four different populations (KORA, Sorbs, HapMap CEU, HapMap TSI). Numbers of remaining markers at each step of pre-processing are presented in bold. [file 1471-2156-12-67-S1.PDF]
