# Supplementary material for: Population-genetic comparison of the Sorbian isolate population in Germany with the German KORA population using genome-wide SNP arrays
Source: BMC Genet. 2011 Jul 28;12:67. doi: 10.1186/1471-2156-12-67 (PMC3199861; doi:10.1186/1471-2156-12-67)
Supplement: Additional file 2 — Derivation of the formula for . [file 1471-2156-12-67-S2.PDF]

Consider a linear model $\mathbf{y} = \alpha + \beta\mathbf{x} + \boldsymbol{\varepsilon}$ with random vectors $\mathbf{y}$ and $\mathbf{x}$ and random errors $\boldsymbol{\varepsilon} \sim \mathrm{N}_n(\mathbf{0}, \sigma^2\mathbf{I})$.
It can be shown that the least square estimator has the following formula[1]

$$\hat{\beta} = \frac{\sum (x-\bar{x})(y-\bar{y})}{\sum (x-\bar{x})^2} \text{ with } \mathrm{E}(\hat{\beta}) = \frac{\mathrm{Cov}(x,y)}{\mathrm{Var}(x)} \text{ and} \quad (1)$$

$$\mathrm{Var}(\hat{\beta}) = \frac{\sigma^2}{\sum (x-\bar{x})^2}. \quad (2)$$

For a SNP with genotypes $\mathbf{s}_1$, we modeled the phenotype $\mathbf{y}$ as followed

$$\mathbf{y} = \beta_1\mathbf{s}_1 + \boldsymbol{\varepsilon}_1. \quad (3)$$

Let $\beta_1 = 1$ and R_s^2 is the proportion of explained variance of y by s_1 then $\boldsymbol{\varepsilon}_1$ is multivariate normal with covariance matrix $\mathrm{Var}(s_1)(\frac{1}{R_s^2} - 1)\mathbf{I}$

$$\boldsymbol{\varepsilon}_1 \sim \mathrm{N}_n(\mathbf{0}, \mathrm{Var}(s_1)(\frac{1}{R_s^2} - 1)\mathbf{I}). \quad (4)$$

For a SNP with genotypes $\mathbf{s}_2$ in maximum LD to the first SNP, we analysed the model

$$\mathbf{y} = \beta_2\mathbf{s}_2 + \boldsymbol{\varepsilon}_2. \quad (5)$$

We are interested in the expected value and variance of $\hat{\beta}_2 \sim \mathrm{N}(\beta_2, \sigma^2_{\hat{\beta}_2})$ in order to calculate the power of the test.

Using (1), for the expected value of $\hat{\beta}_2$ it holds that

$$\mathrm{E}(\hat{\beta}_2) = \frac{\mathrm{Cov}(s_2, y)}{\mathrm{Var}(s_2)}$$

After substituting y with (3), we obtain

$$\mathrm{E}(\hat{\beta}_2) = \frac{\mathrm{Cov}(s_1, s_2)}{\mathrm{Var}(s_2)} = \beta_2. \quad (6)$$

Equation (2) was used in order to calculate the variance of $\hat{\beta}_2$

$$\mathrm{Var}(\hat{\beta}_2) = \frac{\mathrm{Var}(\varepsilon_2)}{\sum (s_2 - \bar{s}_2)^2}$$

The variance of ε_2 can be calculated using (5). One obtains

$$\mathrm{Var}(\varepsilon_2) = \mathrm{Var}(y) - \beta_2{}^2\mathrm{Var}(s_2).$$

Substituting y with (3) we deduce

$$\mathrm{Var}(\varepsilon_2) = \mathrm{Var}(s_1) + \mathrm{Var}(\varepsilon_1) - \beta_2{}^2\mathrm{Var}(s_2).$$

Replacing $\mathrm{Var}(\varepsilon_1)$ by the variance formula given by (4) and β_2 by equation (6), we receive

$$\mathrm{Var}(\varepsilon_2) = \frac{\mathrm{Var}(s_1)}{R_s^2} - \frac{\mathrm{Cov}(s_1, s_2)^2}{\mathrm{Var}(s_2)}.$$

Hence,

$$\mathrm{Var}(\hat{\beta}_2) = \frac{\frac{\mathrm{Var}(s_1)}{R_s^2} - \frac{\mathrm{Cov}(s_1, s_2)^2}{\mathrm{Var}(s_2)}}{\sum (s_2 - \bar{s}_2)^2} = \sigma^2_{\hat{\beta}_2}. \quad (7)$$

Let $z_{\mathrm{pval}/2}$ be the pval/2-quantile of the t-distribution with N-2 degrees of freedom (N is the number of individuals) corresponding to a p-value threshold of a two-sided test of $\hat{\beta}_2$ against the null hypothesis $\beta_2 = 0$ then the power Pwr to reject this null hypothesis under a significance level pval is

$\mathrm{Pwr}_{\mathrm{pval}} = \mathrm{P}(\hat{\beta}_2 < z_{\mathrm{pval}/2}\sigma_{\hat{\beta}_2} \text{ or } \hat{\beta}_2 > -z_{\mathrm{pval}/2}\sigma_{\hat{\beta}_2})$ which can easily be calculated using the expected value and variance of $\hat{\beta}_2$ given by equations (6) and (7).

---

[1]Stuart A, Ord K, Arnold S: **Kendall's Advanced Theory of Statistics**, vol. 2A, 6 edn. London: Wiley; 1999
