# Supplementary material for: Population-genetic comparison of the Sorbian isolate population in Germany with the German KORA population using genome-wide SNP arrays
Source: BMC Genet. 2011 Jul 28;12:67. doi: 10.1186/1471-2156-12-67 (PMC3199861; doi:10.1186/1471-2156-12-67)
Supplement: Additional file 3 — Comparisons of power for Sorbs977 for minimal and maximal heritability of phenotypes. Simulation results of the power for minimal () and maximal (100%) heritability. For the minimal heritability, we present the results of our analytical formula. The values presented in Tables 3 and 4 are displayed in bold. [file 1471-2156-12-67-S3.PDF]

	Explained Variance			
	2%		5%	
p-value treshold	Minimal Heritability	Maximal Heritability	Minimal Heritability	Maximal Heritability
$1x10^{-1}$	99.28	97.8		
$1x10^{-2}$	93.51	89.4		
$1x10^{-3}$	78.67	74.35		
$1x10^{-4}$	57.49	56.2		
**$1x10^{-5}$**	**36.51**	**38.95**		
$1x10^{-6}$			94.93	91.1
**$1x10^{-7}$**			**88.37**	**83.6**
$1x10^{-8}$			78.23	73.7
$1x10^{-9}$			65.21	62.4
$1x10^{-10}$			50.89	50.65
$1x10^{-11}$			37.11	39
$1x10^{-12}$			25.29	28.9
$1x10^{-13}$			16.14	20.75
$1x10^{-14}$			9.67	14.2
