# Supplementary material for: Population-genetic comparison of the Sorbian isolate population in Germany with the German KORA population using genome-wide SNP arrays
Source: BMC Genet. 2011 Jul 28;12:67. doi: 10.1186/1471-2156-12-67 (PMC3199861; doi:10.1186/1471-2156-12-67)
Supplement: Additional file 4 — Variance inflation under relatedness. Comparison of the theoretical variance of the β1-estimator assuming uncorrelated phenotypes (analytical formula ) with the averaged variances over all SNPs of chromosome 22 under a heritability of 100% assuming correlated phenotypes. The standard error of this estimate and the inflation factor are also provided. Sorbs977 are presented in bold due to high inflation of variances of β1-estimates. [file 1471-2156-12-67-S4.PDF]

Explained variance	Population	Expected variance of β-estimator	Empirical mean of variances of β-estimators	Standard error of mean of variances	Variance Inflation
2%	$KORA_{977}$	0.0502	0.0514	$2.5x10^{-3}$	1.023
	**$Sorbs_{977}$**	**0.0502**	**0.0725**	**$7.9x10^{-3}$**	**1.444**
	$KORA_{532}$	0.0923	0.0935	$4.4x10^{-3}$	1.013
	$Sorbs_{532}$	0.0923	0.0954	$4.7x10^{-3}$	1.033
5%	$KORA_{977}$	0.0195	0.0199	$1x10^{-3}$	1.023
	**$Sorbs_{977}$**	**0.0195**	**0.0281**	**$3x10^{-3}$**	**1.442**
	$KORA_{532}$	0.0358	0.0363	$1.7x10^{-3}$	1.014
	$Sorbs_{532}$	0.0358	0.0369	$1.8x10^{-3}$	1.033
