# Supplementary material for: Population-genetic comparison of the Sorbian isolate population in Germany with the German KORA population using genome-wide SNP arrays
Source: BMC Genet. 2011 Jul 28;12:67. doi: 10.1186/1471-2156-12-67 (PMC3199861; doi:10.1186/1471-2156-12-67)
Supplement: Additional file 5 — Simulation results for power under assumption of correlated phenotypes. Heritability was modified between and 100%. Explained variances of the SNP are 2% or 5% with corresponding p-value thresholds of 10-5 and 10-7, respectively. All simulations were performed for KORA977, Sorbs977, KORA532, and Sorbs532. Power distribution is derived using the results of all SNPs of Chromosome 22. [file 1471-2156-12-67-S5.PDF]

Heritability	Explained Variance	p-value treshold	Population	1st Quartile	Median	3rd Quartile
R_s^2	2%	$1x10^{-5}$	$KORA_{977}$	6.7	37.15	48.8
			$Sorbs_{977}$	6.4	36.6	48.9
			$KORA_{532}$	1.2	7.9	11.5
			$Sorbs_{532}$	1.2	8	11.6
	5%	$1x10^{-7}$	$KORA_{977}$	24.75	88.7	95.6
			$Sorbs_{977}$	23.38	88.5	95.6
			$KORA_{532}$	2.8	30	43.3
			$Sorbs_{532}$	2.7	30.3	43.5
20%	2%	$1x10^{-5}$	$KORA_{977}$	6.9	36.9	48.7
			$Sorbs_{977}$	7.17	37.15	48.8
			$KORA_{532}$	1.2	7.9	11.5
			$Sorbs_{532}$	1.2	7.9	11.7
	5%	$1x10^{-7}$	$KORA_{977}$	25	88.8	95.5
			$Sorbs_{977}$	24.17	87.35	95
			$KORA_{532}$	2.7	30	43.2
			$Sorbs_{532}$	2.8	30.6	43.6
40%	2%	$1x10^{-5}$	$KORA_{977}$	6.7	37.3	48.7
			$Sorbs_{977}$	8	37.4	48.9
			$KORA_{532}$	1.2	8	11.5
			$Sorbs_{532}$	1.2	8	11.8
	5%	$1x10^{-7}$	$KORA_{977}$	24.78	88.5	95.4
			$Sorbs_{977}$	24.85	86.5	94.2
			$KORA_{532}$	2.7	30.1	43.1
			$Sorbs_{532}$	2.9	30.1	43.6
60%	2%	$1x10^{-5}$	$KORA_{977}$	6.7	37.15	48.6
			$Sorbs_{977}$	8.6	38	49
			$KORA_{532}$	1.2	7.9	11.5
			$Sorbs_{532}$	1.2	8	11.8
	5%	$1x10^{-7}$	$KORA_{977}$	24.7	88.4	95.3
			$Sorbs_{977}$	25.6	85.4	93.4
			$KORA_{532}$	2.8	29.9	43.1
			$Sorbs_{532}$	2.8	29.9	43.5
80%	2%	$1x10^{-5}$	$KORA_{977}$	6.7	37	48.6
			$Sorbs_{977}$	9.4	38.35	49
			$KORA_{532}$	1.2	7.9	11.5
			$Sorbs_{532}$	1.3	8.1	11.9
	5%	$1x10^{-7}$	$KORA_{977}$	24.87	88.25	95.2
			$Sorbs_{977}$	26.28	84.6	92.7
			$KORA_{532}$	2.73	30.15	43
			$Sorbs_{532}$	2.9	30.3	43.5
100%	2%	$1x10^{-5}$	$KORA_{977}$	6.7	37.1	48.4
			$Sorbs_{977}$	10.08	38.95	48.9
			$KORA_{532}$	1.2	7.8	11.6
			$Sorbs_{532}$	1.3	8.2	11.9
	5%	$1x10^{-7}$	$KORA_{977}$	24.78	88.3	95.12
			$Sorbs_{977}$	27.3	83.6	91.8
			$KORA_{532}$	2.73	29.9	42.9
			$Sorbs_{532}$	2.9	30.4	43.5
