# Supplementary material for: Population-genetic comparison of the Sorbian isolate population in Germany with the German KORA population using genome-wide SNP arrays
Source: BMC Genet. 2011 Jul 28;12:67. doi: 10.1186/1471-2156-12-67 (PMC3199861; doi:10.1186/1471-2156-12-67)
Supplement: Additional file 6 — Additional inbreeding and co-ancestry coefficients. Estimates and standard errors (SE) of inbreeding coefficients FIS and co-ancestry coefficients FST for KORA and Sorbs and different levels of relatedness: without filtering for relatedness (KORA977, Sorbs977), filtering for relatedness > 0.2 (KORA532, Sorbs532), filtering for relatedness > 0.1 (KORA414, Sorbs414). Indices refer to resulting numbers of cases. [file 1471-2156-12-67-S6.PDF]

Population	F-statistic	Estimate	SE
$KORA_{977}$	F_{IS}	0.00117	$2.7x10^{-4}$
$Sorbs_{977}$	F_{IS}	-0.00059	$2.7x10^{-4}$
$KORA_{532}$	F_{IS}	0.00136	$3.5x10^{-4}$
$Sorbs_{532}$	F_{IS}	-0.00015	$3.6x10^{-4}$
$KORA_{414}$	F_{IS}	0.00133	$4.1x10^{-4}$
$Sorbs_{414}$	F_{IS}	-0.00002	$4.2x10^{-4}$
$KORA_{977}$,$Sorbs_{977}$	F_{ST}	0.00342	$5.4x10^{-5}$
$KORA_{532}$,$Sorbs_{532}$	F_{ST}	0.00294	$6.7x10^{-5}$
$KORA_{414}$,$Sorbs_{414}$	F_{ST}	0.00273	$7.4x10^{-5}$
